# A Rare Case of Brain Manifestation of Manganese Toxicity and Its Management: A Case Report and Literature Review

**DOI:** 10.7759/cureus.98567

**Published:** 2025-12-06

**Authors:** Maulinkumar N Patel, Aastha Arunkumar Patel, Chandan Alenahalli Narayana, Rutuja Tere, Dhruvil Vinaybhai Patel, Saniah Khan, Zenia Elavia, Mansi Singh

**Affiliations:** 1 Epidemiology and Biostatistics, The University of Texas Health Science Center at Houston, Houston, USA; 2 Internal Medicine, GMERS Medical College and Hospital, Gandhinagar, Gandhinagar, IND; 3 College of Medicine, Bangalore Medical College and Research Institute, Bengaluru, IND; 4 Internal Medicine, Lokmanya Tilak Municipal Medical College and General Hospital, Mumbai, IND; 5 Internal Medicine, King Edward Medical University, Lahore, PAK; 6 College of Medicine, Dr. D. Y. Patil Medical College, Hospital and Research Centre, Pune, IND; 7 Medicine, Bogomolets National Medical University, Kyiv, UKR

**Keywords:** acute on chronic liver disease, confabulation, continuous chelation, globus pallidus lesions, manganese toxicity

## Abstract

Manganese (Mn) toxicity is a rare but perplexing condition, often difficult to diagnose due to its unusual clinical presentation. Mn toxicity's neurological symptoms can be nonspecific, making early diagnosis crucial. The unique association between chronic liver disease and Mn toxicity highlights the need for increased clinical awareness and further research. We present a case of a five-year-old Asian male child with chronic liver disease who presented with neurological symptoms due to brain Mn deposition and its management. Notably, Mn exhibits a propensity for preferential accumulation in the basal ganglia, specifically targeting dopamine-producing (DAergic) neurons. The child's response to interventions, including a reduction in Mn deposition in the brain using chelating therapy, underscores the significance of early recognition and comprehensive management in addressing this condition. This case report delves into an exceptional instance of Mn toxicity, emphasizing the significance of early recognition and interdisciplinary intervention.

## Introduction

Manganese (Mn), the fifth most abundant metal and 12th overall in the earth’s crust, is an essential trace element vital for enzymatic and metabolic processes in humans, with dietary intake typically meeting daily requirements [1,2]. However, excessive Mn can cause neurotoxicity, particularly in individuals with impaired biliary excretion, such as those with chronic liver disease [3,4]. Accumulation may result from defective transporter function, environmental exposure (air, water, food), or total parenteral nutrition (TPN) [3]. In liver dysfunction, Mn deposition in the basal ganglia can lead to acquired hepatocerebral degeneration, affecting 1-2% of cases and causing Parkinsonism, dystonia, ataxia, and cognitive deficits [4]. Pediatric cases, though rare, are reported in cholestatic disorders, presenting with developmental delays, hyperactivity, and motor impairments, often exacerbated by TPN or genetic mutations like SLC30A10, which increase hypermanganesemia risk [3-5].

This report presents a rare case of Mn neurotoxicity in a five-year-old boy with chronic liver disease, highlighting the need for early recognition and multidisciplinary management. Characteristic imaging findings, such as bilateral, symmetrical T1 hyperintensities in the globus pallidus and anterior midbrain, suggested Mn deposition, aligning with paramagnetic effects seen in pediatric hepatic failure [5]. Through this case and a literature review [6-8], we address diagnostic challenges, clinical significance, and therapeutic strategies for Mn toxicity, aiming to raise awareness of this under-recognized condition and emphasize collaborative interventions for better outcomes. The objective of this case report is to describe a rare presentation of Mn neurotoxicity in a five-year-old child with chronic liver disease, including the clinical presentation, diagnostic pathway, and treatment response. This report emphasizes the importance of early recognition and a multidisciplinary management approach to prevent irreversible neurological sequelae.

## Case presentation

A five-year-old Asian male child with a known history of chronic liver disease presented with a seven-day history of mental confabulation. The patient's neurological manifestations were multifaceted and indicative of basal ganglia involvement due to Mn deposition. Initially, he exhibited prominent mental confabulation, characterized by the fabrication of false memories and incoherent narratives, often filling gaps in recall with invented details. This was accompanied by behavioral changes, including irritability, hyperactivity, and emotional lability, such as frequent outbursts and difficulty maintaining attention during interactions. Motor symptoms included mild dystonia, manifested as involuntary muscle contractions leading to abnormal posturing of the limbs, subtle tremors in the hands, and ataxic gait with unsteadiness while walking. Cognitive impairments were evident, with deficits in executive function, such as poor problem-solving and impaired short-term memory. These symptoms align with reported pediatric cases of Mn neurotoxicity in cholestatic liver disease, where basal ganglia accumulation leads to extrapyramidal and neuropsychiatric disturbances. Prior to treatment, the child's behavior was markedly disruptive; he was unable to engage in age-appropriate play, showed resistance to routine activities, and required constant supervision due to safety concerns from motor instability and confusion.

**Figure 1:**
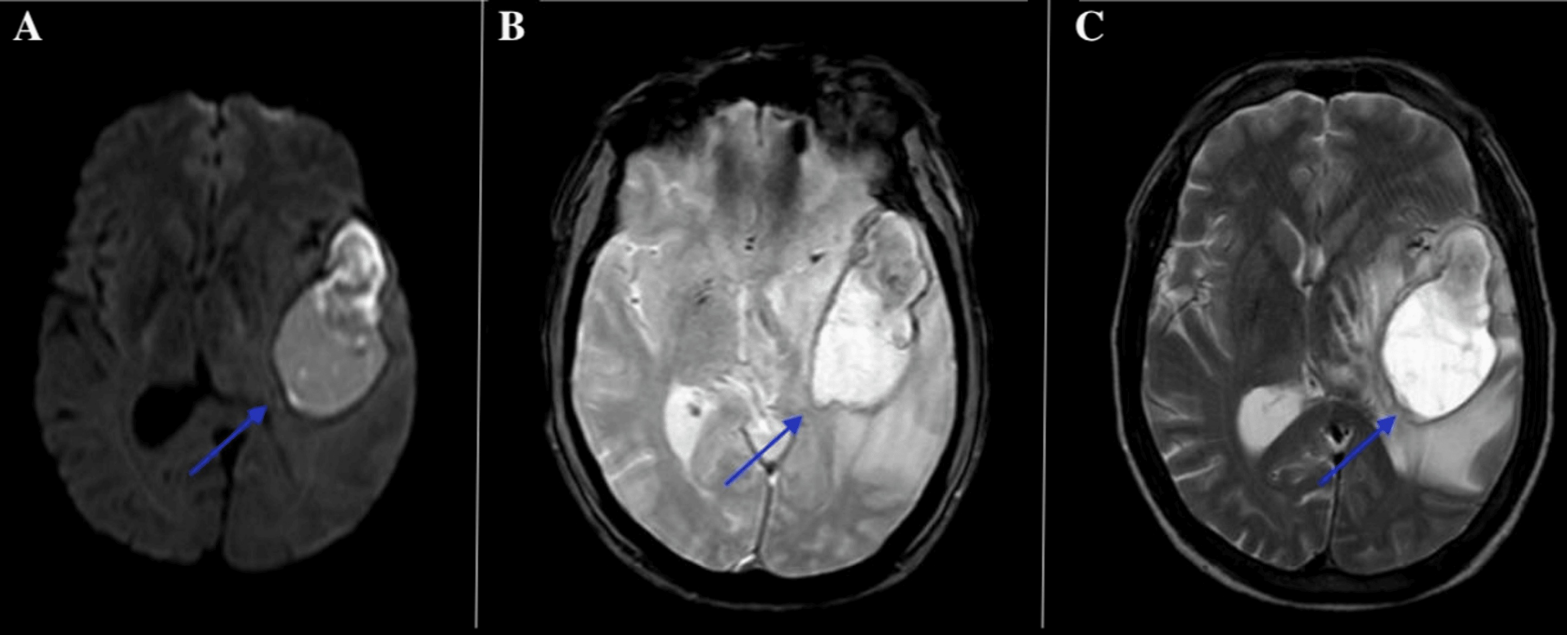
MRI brain images (axial sections) showing (A) diffusion-weighted imaging, (B) susceptibility-weighted imaging, and (C) T2-weighted imaging. The blue arrows indicate a well-defined hyperintense lesion involving the right thalamus and adjacent structures, consistent with an acute hemorrhagic lesion.

Initial neurological evaluation was complemented by magnetic resonance imaging (MRI). A plain T1-weighted sequence revealed bilateral, symmetrical hyperintensities in the globus pallidus and anterior midbrain (Figure 1). These findings were strongly suggestive of Mn deposition and correlated well with the patient’s neurological manifestations.

Based on dietary evaluation and clinical history, the likely source of Mn exposure was prolonged intake of a Mn-fortified pediatric formula used for nutritional supplementation. No other significant environmental or parenteral sources were identified. Given the patient’s chronic liver disease and the characteristic neuroimaging pattern, Mn toxicity was considered the most likely diagnosis.

A multidisciplinary management plan was initiated, involving specialists from neurology, hepatology, and toxicology. The therapeutic approach focused on reducing Mn levels, optimizing liver function, and providing neuroprotective support. First, efforts were made to eliminate ongoing Mn exposure; although specific sources were not detailed, this included reviewing and adjusting any dietary or supplemental intakes, such as those potentially from TPN if applicable. Chelation therapy was promptly started with intravenous calcium disodium ethylenediaminetetraacetic acid (CaNa2EDTA) at a dose of 20 mg/kg/day for five days, repeated in cycles based on blood Mn monitoring, to enhance urinary excretion of the metal. Concurrently, the patient received oral vitamin E supplementation (400 IU daily) to mitigate oxidative stress and neuronal damage induced by Mn. Liver function optimization involved hepatoprotective measures, including ursodeoxycholic acid to improve biliary flow and reduce cholestasis, alongside supportive care such as nutritional therapy tailored to minimize further Mn intake. Clinical progress was closely monitored with serial neurological assessments, blood Mn levels every two weeks, and follow-up MRI at three-month intervals. Consultation with a poison control center was sought to refine the regimen, ensuring safety in this pediatric context.

Over time, the patient demonstrated significant neurological improvement. Within four weeks of initiating treatment, blood Mn levels decreased from elevated baseline to near-normal ranges (Table 1), correlating with behavioral changes. Post-treatment, the child's confabulation resolved completely, replaced by coherent speech and accurate memory recall. Hyperactivity and irritability diminished markedly; he became more engaged in play, showed improved attention span, and exhibited stable emotional responses. Motor symptoms improved, with resolution of dystonia and tremors, allowing normal gait and fine motor activities. Repeat MRI at three months showed a notable reduction in T1 hyperintensities in the globus pallidus and midbrain, confirming decreased Mn deposition. Overall, his condition improved considerably, enabling a return to preschool activities with minimal residual deficits, underscoring the efficacy of early intervention.

**Table 1:** Changes in whole blood manganese concentration following intervention. All values are expressed in nmol/L. The normal pediatric reference interval is 73-273 nmol/L. A decline toward this range correlates with clinical improvement.

Parameter	Reference Range	Pre-treatment Value	Post-treatment Value (Three-Month Follow-Up)
Whole Blood Manganese	72.8-218.5 nmol/L	428 nmol/L	176 nmol/L

Published pediatric reports of Mn toxicity associated with chronic liver disease remain scarce, and most available literature describes adult cases of acquired hepatocerebral degeneration. Consistent with previous reports, this patient demonstrated symmetrical T1 hyperintensity in the globus pallidus correlating with neurological symptoms. However, in contrast to several cases where delayed diagnosis resulted in persistent deficits, this patient showed marked neurological and radiological improvement following early chelation and liver-directed management. This case, therefore, reinforces emerging evidence that timely intervention may lead to reversibility of Mn-related neurotoxicity.

## Discussion

Mn is an essential trace metal with important physiological roles, particularly as a cofactor in enzymatic processes. In children aged four to eight years, the recommended daily intake is approximately 1.5 mg/day [9]. It is involved in critical metabolic pathways, including gluconeogenesis through activation of pyruvate carboxylase, the Krebs cycle via isocitrate dehydrogenase, and the antioxidant defense system as a component of superoxide dismutase (SOD). Within the central nervous system (CNS), Mn is a vital cofactor for glutamine synthetase, predominantly located in astrocytes [10]. These functions highlight Mn's indispensability for normal neurological development, bone formation, and metabolic homeostasis in growing children.

Despite its nutritional significance, Mn becomes neurotoxic when accumulated in excessive amounts, leading to manganism - a syndrome characterized by psychiatric disturbances, motor impairments, and cognitive deficits [11]. The mechanisms underlying Mn neurotoxicity include mitochondrial dysfunction, oxidative stress via reactive oxygen species generation, protein misfolding and aggregation, and disruption of neurotransmitter systems, particularly dopaminergic signaling [12,13]. In pediatric populations, symptoms often manifest as subtler neuropsychiatric issues such as hyperactivity, irritability, developmental delays, and emotional lability, differing from the more pronounced Parkinsonian features in adults [14]. Children with underlying liver disease or those on long-term TPN are at particular risk of Mn overload due to impaired biliary excretion - the primary elimination route - and potential excess supplementation in TPN solutions [15]. Reports have also highlighted elevated Mn concentrations in infant formulas, further contributing to the risk of hypermanganesemia in formula-fed infants [16].

Recent advances have identified recessive mutations in the SLC30A10 gene as a cause of hereditary Mn overload. This transporter defect impairs Mn efflux, leading to a syndrome characterized by hepatic cirrhosis, dystonia, polycythemia, and basal ganglia Mn deposition, evident on MRI even in the absence of environmental exposure [8,17,18]. Similarly, mutations in SLC39A14, another Mn transporter, have been associated with childhood-onset Parkinsonism-dystonia and hypermanganesemia [18].

The hallmark neuroimaging feature of Mn toxicity is bilateral, symmetrical T1 hyperintensity in the basal ganglia, especially the globus pallidus, striatum, and substantia nigra, due to the paramagnetic properties of Mn [5,19]. In the present case, these imaging features, combined with the clinical context of chronic liver disease, were strongly suggestive of Mn toxicity as the cause of neurological symptoms, aligning with reported cases in pediatric hepatic failure [20].

Characteristics of Mn and iron have been implicated in neurotoxicity, with competitive interactions at transport proteins and non-redox domains, exacerbating oxidative pathways [21,22]. Consequently, compounds with iron-chelating properties or antioxidant activity, such as polyphenols, have been explored for therapeutic benefit in mitigating Mn-induced damage [21-24]. In addition, vitamin E supplementation has been shown to exert neuroprotective effects against Mn-induced oxidative stress by scavenging free radicals and preserving neuronal integrity [25,26].

In this case, a multidisciplinary approach involving chelation therapy and vitamin E supplementation resulted in marked clinical and radiological improvement, with resolution of confabulation, hyperactivity, and motor symptoms, alongside reduced T1 hyperintensities on follow-up MRI. This outcome underscores the efficacy of early diagnosis and prompt intervention in reversing Mn neurotoxicity, particularly in pediatric patients, where neuroplasticity may enhance recovery [27]. The significance of these findings lies in the relative rarity of symptomatic Mn neurotoxicity in children with chronic liver disease without overt genetic mutations or prolonged TPN, as most literature focuses on occupational exposures in adults or hereditary cases [28,29]. This report adds to the limited pediatric case series, emphasizing the diagnostic value of MRI as a non-invasive biomarker for Mn deposition and the potential for favorable outcomes with tailored chelation and supportive therapies [30,31]. Clinically, it highlights the need for routine Mn monitoring in at-risk children, adjustment of nutritional regimens to prevent overload, and awareness of atypical presentations to improve prognosis and prevent irreversible neurological sequelae.

## Conclusions

The presented case of Mn toxicity in a five-year-old child with chronic liver disease underscores the unique neuropathological mechanism of Mn, particularly its predilection for accumulating in the basal ganglia and affecting dopaminergic neurons. The marked clinical improvement and radiological reduction in Mn deposition following timely intervention highlight the critical importance of early recognition and multidisciplinary management. This case adds to the limited body of literature on pediatric Mn toxicity and reinforces the need for heightened clinical vigilance, especially in children with chronic liver disease or other risk factors. Tailored treatment strategies, including chelation therapy and neuroprotective supplementation, remain essential for optimizing outcomes in this rare but potentially reversible condition. This case highlights that Mn neurotoxicity, although rare, is a reversible and clinically important complication of pediatric chronic liver disease, and early recognition supported by MRI and coordinated multidisciplinary management can significantly improve outcomes.
